# Glycan Recognition in Human Norovirus Infections

**DOI:** 10.3390/v13102066

**Published:** 2021-10-14

**Authors:** Victoria R. Tenge, Liya Hu, B. V. Venkataram Prasad, Göran Larson, Robert L. Atmar, Mary K. Estes, Sasirekha Ramani

**Affiliations:** 1 Department of Molecular Virology and Microbiology, Baylor College of Medicine, Houston, TX 77030, USA; victoria.tenge@bcm.edu (V.R.T.); vprasad@bcm.edu (B.V.V.P.); ratmar@bcm.edu (R.L.A.); mestes@bcm.edu (M.K.E.); 2 Verna and Marrs McLean Department of Biochemistry and Molecular Biology, Baylor College of Medicine, Houston, TX 77030, USA; lhu@bcm.edu; 3 Department of Laboratory Medicine, University of Gothenburg, SE 413 45 Gothenburg, Sweden; goran.larson@clinchem.gu.se; 4 Department of Medicine, Baylor College of Medicine, Houston, TX 77030, USA

**Keywords:** human noroviruses, histo-blood group antigens, glycoconjugates, human intestinal enteroids/organoids, structure, host–virus interactions

## Abstract

Recognition of cell-surface glycans is an important step in the attachment of several viruses to susceptible host cells. The molecular basis of glycan interactions and their functional consequences are well studied for human norovirus (HuNoV), an important gastrointestinal pathogen. Histo-blood group antigens (HBGAs), a family of fucosylated carbohydrate structures that are present on the cell surface, are utilized by HuNoVs to initially bind to cells. In this review, we describe the discovery of HBGAs as genetic susceptibility factors for HuNoV infection and review biochemical and structural studies investigating HuNoV binding to different HBGA glycans. Recently, human intestinal enteroids (HIEs) were developed as a laboratory cultivation system for HuNoV. We review how the use of this novel culture system has confirmed that fucosylated HBGAs are necessary and sufficient for infection by several HuNoV strains, describe mechanisms of antibody-mediated neutralization of infection that involve blocking of HuNoV binding to HBGAs, and discuss the potential for using the HIE model to answer unresolved questions on viral interactions with HBGAs and other glycans.

## 1. Introduction

The surfaces of cells are heavily decorated with glycan structures ranging from simple monosaccharides to complex sugars and glycoconjugates, which vary in branching, linkages, and orientations. The specific glycan structures differ between species and are determined by tissue expression of glycosyltransferases, enzymes that transfer sugar residues to a growing oligosaccharide chain [1,2,3]. Gut microbiota can also degrade and modify endogenous and dietary glycan structures, altering their distribution and availability in the intestines [4,5]. Pathogens exploit host glycans for initial cell recognition and attachment. This is particularly well studied for enteric pathogens such as *Helicobacter pylori*, human norovirus (HuNoV), and human rotavirus. This review focuses on HuNoV–glycan interactions as these have been investigated from many different perspectives including human infection data from epidemiological and controlled experimental infection studies, in vitro biochemical and structural studies, and ex vivo infection experiments.

**Figure 1:**
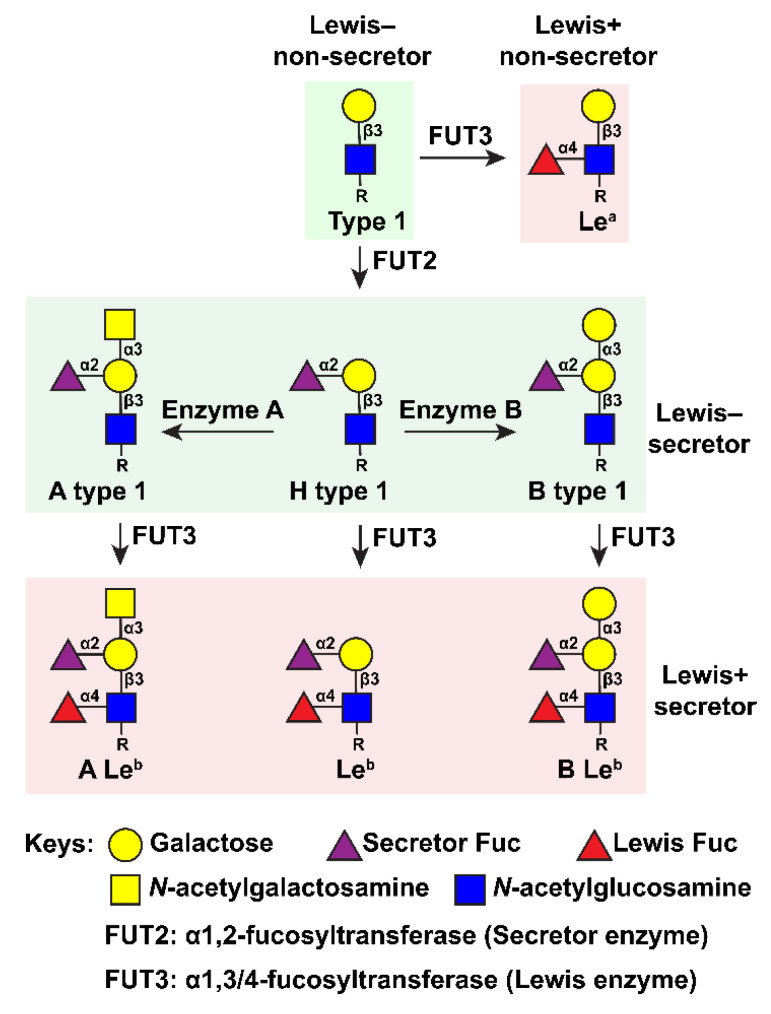
HBGA-chain modification pathways on the type-1 structure. Adapted from Hu et al., 2018 under the terms of the Creative Commons Attribution 4.0 International license (https://creativecommons.org/licenses/by/4.0/) [23]. Accessed on 25 August 2021.

HuNoVs recognize a family of fucosylated glycans termed histo-blood group antigens (HBGAs), and susceptibility to infection is determined by host genetic capability to generate these structures [6,7,8,9,10]. HBGAs are formed through a series of monosaccharide additions by various glycosyltransferases to mainly type-1 and type-2 precursor glycans. Fucosyltransferase 1 (FUT1) and fucosyltransferase 2 (FUT2) may both add fucose (Fuc) in an α1,2-linkage to a terminal galactose (β-Gal) on the precursor glycan (β-galactose-N-acetyl-glucosamine), forming H-type HBGA structures. The tissue distribution of glycans formed by these two transferases is quite different, with FUT1 products mainly expressed in erythrocyte progenitor cells and FUT2 products mainly expressed in epithelial tissues, such as in the intestine [11]. FUT1 preferentially acts on the type-2 precursor (Galβ1,4-linkage) and FUT2 on the type-1 precursor (Galβ1,3-linkage). FUT2 is encoded by the secretor gene (*FUT2, Se*) and individuals expressing a functional FUT2 enzyme are referred to as secretors. The *Se* gene is dominant and specific mutations can render it partially (*Se_w_*) or completely non-functional (*se*); persons who are homozygous for *se* are referred to as non-secretors [12,13,14,15,16,17]. Non-secretors are resistant to infection by many HuNoV strains, with some exceptions [12,18,19,20,21]. However, gut bacteria such as *H. pylori* expressing the virulence factor CagA can modify glycans, leading to detection of fucose in non-secretor tissues, and can thus influence susceptibility to HuNoV [4]. In addition to the *Se* gene, the *Lewis* (*FUT3*, *Le*) gene encoding the FUT3 enzyme, as well as the *ABO* gene, can also influence susceptibility to HuNoV infection [20]. FUT3 adds a fucose residue in α1,3- or α1,4-linkage to the N-acetylglucosamine (GlcNAc) of either precursor glycan to generate Lewis a or Lewis x (Le^a/x^), or to the H-type structures to generate Lewis b or Lewis y (Le^b/y^) glycans depending on whether the precursors are of type-1 or type-2. The *ABO* locus encodes three alleles. The *A* and *B* alleles encode for the A and B enzymes while the *O* allele leads to an inactive enzyme and the H phenotype. Enzyme A and B add either an N-acetylgalactosamine (GalNAc) or a Gal residue in α1,3-positions, respectively, to the β-Gal moiety of the H-type precursor structures. These A or B structures may be further modified by FUT3 to generate A or B Lewis b/y (A Le^b/y^, B Le^b/y^) glycans [22]. The fucosylated type-1 glycan structures formed by the activity of FUT2, FUT3, and A/B enzymes are shown in Figure 1.

In this paper, we review data on the discovery of HBGAs as genetic susceptibility factors for HuNoV infection, and the structural and functional basis for HuNoV interactions with specific glycans. We also describe how human intestinal enteroids (HIEs), a new ex vivo model for HuNoV replication, reflect differences in host genetic susceptibility to this virus, and discuss how HIEs can be used to gain a deeper understanding of the human glycome. 

## 2. Norovirus Disease Burden, Classification, and Epidemiology

HuNoV is a leading cause of foodborne, epidemic, and acute gastroenteritis worldwide. The virus was first identified after a 1968 outbreak of transmissible vomiting and diarrhea at an elementary school in Norwalk, Ohio [24]. In countries where rotavirus vaccines are effective, HuNoV has replaced rotavirus as the leading cause of viral gastroenteritis in children under the age of 5 years [25,26,27,28,29]. Symptoms of HuNoV-induced disease include vomiting, diarrhea, myalgia, abdominal pain, cramping, fever, chills, headache, and fatigue. Adverse outcomes and prolonged disease are more likely to occur in young children, the immunocompromised, and the elderly [30,31,32]. In addition to health consequences, the economic cost of HuNoV is vast, with an estimated $60 billion spent annually in direct healthcare costs and indirect loss of productivity costs globally [33]. Despite their medical relevance, there was no cell-culture system for the laboratory cultivation of HuNoVs for nearly 50 years after their discovery. Nonetheless, advances were made through genetic, biochemical, structural, and epidemiological studies as well as controlled human experimental infections. 

HuNoVs are classified in the genus Norovirus, part of the family *Caliciviridae* that contains 10 other genera (*Lagovirus*, *Nebovirus*, *Recovirus*, *Sapovirus*, *Valovirus*, *Vesivirus*, *Bavovirus*, *Nacovirus*, *Minovirus*, and *Salovirus*) and several other unclassified caliciviruses [34]. The *Caliciviridae* are non-enveloped viruses with a linear, single-stranded, positive-sense RNA genome encoding for non-structural proteins (ORF1), and structural proteins VP1 (ORF2) and VP2 (ORF3). Characterization of many strains circulating in humans has shown that these viruses are genetically and antigenically diverse. To classify norovirus strains, the amino-acid sequence of VP1 is used to form phylogenic clusters referred to as genogroups and further subgrouped into genotypes [35]. Currently, there are 10 established, and 2 tentative genogroups (GI-GX, GNA1, GNA2), of which GI, GII, GIV, GVIII, and GIX contain viruses that infect humans. The prototype HuNoV, Norwalk virus, is in genogroup I and is genotype one, which is written as GI.1. GI contains genotypes GI.1-GI.9 and GII contains GII.1-GII.27 and two tentative genotypes [35]. Genotypes can be further subdivided into variants. This is best recognized for GII.4, the predominant genotype of HuNoV worldwide. Several minor variants of GII.4 have been described, and until 2012, a new pandemic variant of GII.4 (e.g., GII.4 Sydney_2012) emerged every 2–5 years, replacing the previously dominant variant. Thus, from 1996 through 2012, six GII.4 variants (US95/96, Farmington_Hills_2002, Hunter_2004, Den_Haag_2006, New_Orleans_2009, and Sydney_2012) caused HuNoV pandemics [36]. The GII.4 variants described in this review are summarized in Table 1.

**Figure 2:**
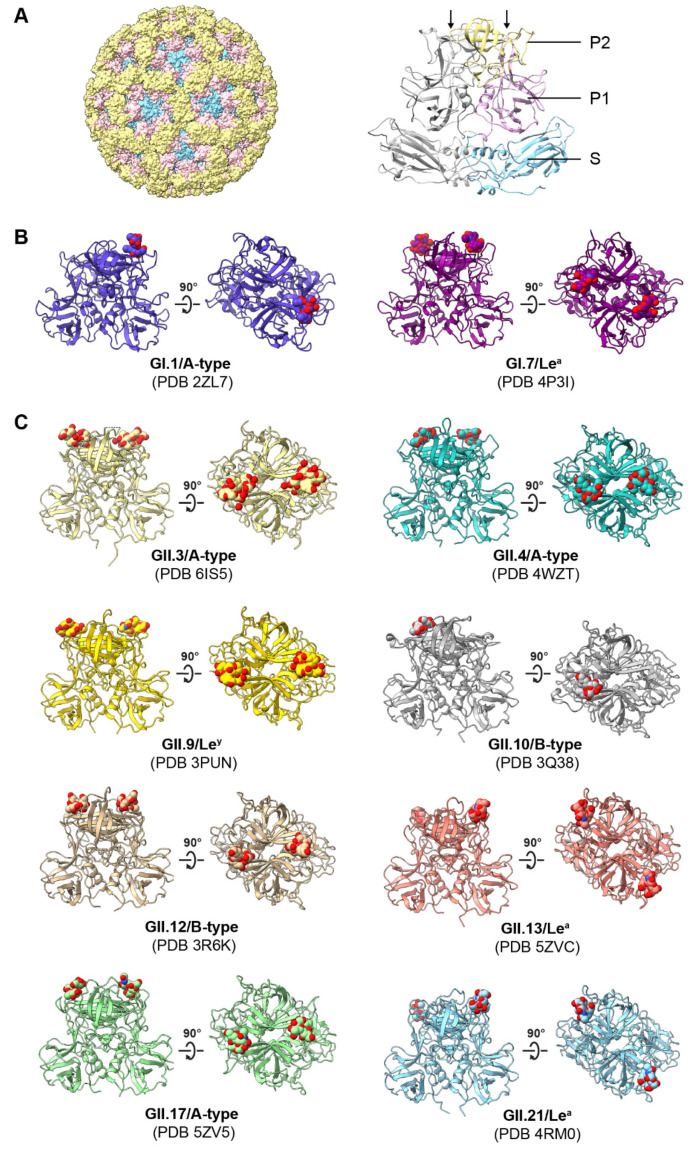
HBGAs bind to HuNoV P domain. (**A**) Crystal structure of the Norwalk virus-like particle (left) comprised of 90 VP1 dimers (right) (PDB ID: 1IHM). The S domain, P1, and P2 subdomains are colored in blue, pink, and yellow, respectively. The second chain of the VP1 dimer is shown in gray. Arrows point to the P-loops in P2. (**B**,**C**) HBGA binding sites located on P domain dimers of GI and GII HuNoVs. The P domains (ribbon representation) and HBGAs (spheres) are shown as side views of the P domain dimers or rotated 90 degrees to show a top view. HBGA carbon coloration matches the ribbon diagram and heteroatoms are colored by element: oxygen, red; nitrogen, blue.

HuNoV capsids are composed of the major structural protein VP1 and the minor structural protein VP2. VP1 contains two domains: the protruding (P) and shell (S) domains. The P domain can be further divided into two subdomains P1 and P2 (Figure 2A). Recombinant VP1 and VP2 will spontaneously assemble into virus-like particles (VLPs) that recapitulate the virus antigenically and morphologically [54,55]. The first HuNoV structure was solved in 1999 and was of a Norwalk VLP. This structure revealed that 90 VP1 dimers form a T = 3 capsid where the S domains contain the core structure surrounding the genome and the P domains create the arch of the cup or calyx-like structures from which the caliciviruses name was derived [56]. The use of VLPs derived from different HuNoV strains has been a critical tool for delineating the importance of HBGAs in HuNoV binding to host tissues and cells. 

## 3. Early Discoveries on the Genetic Basis of Glycan Involvement in HuNoV Infection

Early observations from controlled human-infection trials revealed genetic differences in susceptibility to HuNoVs. A challenge study with Norwalk virus fecal filtrate 8FIIa indicated the potential for genetic resistance to infection when half of the challenged individuals (6/12) remained asymptomatic and failed to develop a virus-specific antibody response, as determined by immunoelectron microscopy after two challenge infections ~2–4 years apart [57]. In vitro studies on a related animal calicivirus and additional human infection studies provided evidence for a host genetic capability to produce HBGA glycans as a factor responsible for susceptibility and resistance to HuNoV. The rabbit hemorrhagic disease virus (RHDV) was able to agglutinate human erythrocytes expressing ABH antigens but not those of the rare Bombay phenotype, which completely lack these glycans, or cord-blood samples with low levels of ABH expression [58]. Furthermore, saliva from a secretor but not a non-secretor, inhibited RHDV agglutination of erythrocytes [58]. Subsequently, it was shown that persons with O blood group were significantly more likely to be infected with Norwalk virus and those with B-blood-group had a reduced risk of infection [59]. A second human challenge trial where secretor status of participants was analyzed determined that non-secretor individuals were not infected after challenge with Norwalk virus, while the majority of secretors were infected [10]. Examination of the salivary IgA response from secretors in this study showed two patterns; persons who never shed virus or did not experience symptoms had an early IgA response, indicative of previous exposure, while others had a delayed IgA response, suggesting a primary immune response [10]. Secretor-status analysis using serum samples from the original challenge study, that correlated Norwalk infection with the O blood group, revealed all infected individuals were secretors and that the uninfected individuals were primarily non-secretors and one B-type secretor [18,59]. 

Supporting the observations from controlled human-infection trials, biochemical studies determined that HuNoVs utilize HBGAs as attachment factors. Norwalk VLPs were able to bind to duodenal tissue or saliva from secretors and these interactions were strongly inhibited by antibodies against H-type-1 or incubation with H-type-3 antigens [9]. Hemagglutination assays similar to those that identified an influenza glycan receptor (α2,6 sialic acid), performed in the cold (4 °C), and at low pH, revealed that Norwalk VLPs hemagglutinate red blood cells (RBCs) from all O, A, and AB groups tested but only from a subset of B RBCs [60]. This study also showed that RBCs lacking H-type-2, such as those from Bombay individuals, could not be hemagglutinated by Norwalk VLPs and that VLPs could bind to synthetic Le^b^, H-type-2, and Le^y^ carbohydrates, all of which can be found in the intestine [60]. Norwalk VLPs were also shown to interact with CHO cells expressing FTB (the rat homolog of FUT2), and Caco-2 cells which express FUT2 were also able to internalize VLPs [9]. Since human HBGA phenotypes are affected by several genes coding for various glycosyltransferases often working sequentially, an important finding was the direct genetic linkage of HuNoV GII infection and inactivating mutations in the human FUT2, but not in the human FUT3 gene [12,14,61,62]. Since then, this genetic linkage has been confirmed in several populations, with a wide variety of completely or partially inactivating mutations in the FUT2 gene [20]. Together, all these studies indicated the importance of HBGAs in HuNoV attachment to host cells and prompted further investigation into their role in infection.

## 4. Norovirus–Glycan Binding Specificity and Structural Interactions

### 4.1. Binding Patterns to HBGAs and Related Glycans

In addition to RBCs and epithelial cells, HBGAs are present in mucosal secretions such as saliva. Therefore, saliva binding assays have been used to characterize HuNoV–HBGA interactions. Saliva samples from adults, characterized to confirm HBGA expression, were used to coat microtiter plates in enzyme immunoassays and then bound with VLPs derived from GI or GII HuNoVs. In total, seven distinct binding patterns emerged (Table 2): (1) two US95/96-variant GII.4 VLPs (VA387 and GrV) bound to saliva from A/B/H individuals, (2) GII.5 (MOH) and two GII.3 VLPs (MxV and PiV) bound A/B saliva but not H-type or non-secretor saliva, (3) GII.2 (BUDS) bound primarily A saliva, (4) Norwalk and GI.2 (C59) bound A/H saliva but the binding of C59 was weak [39,40], (5) GI.8 (Boxer) and GII.9 (VA207) were capable of binding strongly to saliva from non-secretor individuals and to A/H saliva [39,40], (6) GII.21 (OIF) also bound to saliva from non-secretors; however, this VLP only bound weakly to H and not to A saliva [40], and finally, (7) GI.3s (DSV and VA115) and GII.1 (Hawaii virus, HV) did not bind to any saliva samples tested. Of the three strains that could bind to non-secretor saliva, VA207 but not Boxer or OIF VLP binding to Le^a^ and Le^x^ saliva was reduced by blocking with antibodies against Le^a^ and Le^x^, respectively. Additionally, the binding of VA207 and Boxer to Le^b^ and Le^y^ saliva was blocked with antibodies against Le^b^ and Le^y^, respectively [40]. A limitation to these saliva binding assays is that saliva could contain other non-ABH and non-Le glycans that may also influence VLP binding.

Norwalk and Dijon (US95/96 GII.4 variant) VLPs bind to lipid bilayer vesicles incorporated with H-type-1 but not Le^a^ glycosphingolipids (GSLs) [37]. Altering the percentage of H-type-1 GSLs in the lipid bilayer demonstrated that Dijon VLPs required a higher percentage of H-type-1 in the bilayer than Norwalk VLPs for binding. This suggests Dijon VLPs have a lower affinity for H-type-1 GSLs and may require interactions with more GSL molecules to attach to a lipid bilayer [37]. Taken together, there are strain-specific differences in HuNoV binding to different HBGA structures presented both on glycoproteins and on glycolipids. 

HuNoV VLPs are also capable of binding to HBGA-like molecules present in other animal species. Norwalk VLPs can bind to porcine gastric mucin (PGM), and incubation with PGM blocks the binding of VLPs to HBGAs in enzyme immunoassays and of VLP binding to secretor Caco-2 cells as detected by immunofluorescence using an antibody against the VLP [63]. Pig intestinal tissues express A and H antigen and the ability of HuNoVs to bind to porcine HBGA is supported by the binding of Norwalk, MD145 (GII.4 Camberwell variant from 1987), HS66 (US95/96 GII.4 variant), HV, and Toronto virus (TV, GII.3) to duodenal tissue from A- and H-expressing pigs [41]. However, antibodies against A and H did not reduce, or only partially reduced, the binding of VLPs, indicating the VLPs can bind also to other antigens found in the porcine intestine [41]. The B antigen glycosyl transferase can convert the H antigen of PGM to B antigen and this allows a B-recognizing VLP, GII.12, to bind to PGM [64].

### 4.2. Structural Basis of HuNoV–HBGA Interactions

Structure–function studies of the HuNoV P domain have focused primarily on three main sites of interest: HBGA binding sites, bile-acid binding sites (discussed in Section 5), and antibody binding sites (discussed in Section 6). Crystal structures of the HuNoV P domain in complex with HBGA have been determined for two GI and eight GII genotypes (Figure 2B,C) and highlight differences in HBGA binding sites between genogroups. Norwalk virus P domain dimers crystallized in complex with H-type-1 HBGA reveal a shallow surface-exposed binding pocket at the top of the P domain, away from the dimer interface where the α-Fuc and β-Gal of the H-type chain are anchored to the binding site [65]. When A-type antigen is instead present, the terminal GalNAc added to the β-Gal replaces the β-Gal in the binding pocket with α-Fuc. Crystal structures of a GII.4 (Hunter_2004) P domain (TCH05 isolate) showed binding to HBGA in a pocket also on the top of the P domain but at the dimer interface and through interaction with terminal α-Fuc of HBGAs [46]. The HBGA binding pocket is similar to GII.4 for GII.10 and GII.17 [66,67]. More recent GII.4 variants (2001–current, including Hunter_2004) have a single amino-acid insertion in a flexible loop proximal to the HBGA binding pocket capable of supporting interaction with the Lewis fucose residue, permitting Le^b^ binding [46]. The expanded capability of recent GII.4 variants to bind additional HBGA glycans may explain the dominance of this genotype. Among GI viruses, the overall HBGA P domain structure and HBGA binding site are conserved; however, flexible loops that make up the P2 region of the P domain vary and this sequence variation leads to variable HBGA binding among different strains, including binding to non-secretor HBGAs [68]. For example, compared to GI.1, GI.2 and GI.7 have a longer P2 loop (referred to as the P-loop, arrows Figure 2A) that can interact with the Lewis fucose residue on Le^a/x^, whereas the P-loop of GI.1 cannot interact [68]. 

Structures of HuNoVs in complex with human milk oligosaccharides (HMOs) have also been resolved. Breast milk contains several unconjugated HMOs that are similar in structure to HBGAs. Specifically, 2′-fucosyllactose (2′-FL) and 3-fucosyllactose (3-FL) are similar to H-type-2 and Le^x^ antigens, respectively [69]. HMOs are protective against infection by viral and bacterial pathogens that recognize fucosylated glycans, such as in the case of *Campylobacter jejuni* where the infection is blocked by secretor HMOs that act as decoy receptors [70,71]. 2′-FL and 3-FL can bind GII.10 HuNoV in the HBGA binding pocket, suggesting a potential to act as decoy receptors against norovirus infection [72]. 2′-FL can also bind to the HBGA binding pockets on an additional GII HuNoV, GII.17, and to the HBGA binding pocket of GI.1 [67]. Pretreatment of GII.10, GII.17, and GI.1 VLPs with 2′-FL inhibits binding to A-blood-group saliva and PGM in ELISAs [67,72]. Despite variation in the HBGA binding pockets among HuNoV strains, these results suggest that it may be possible to target the HBGA binding pocket as a broad antiviral strategy. 

## 5. HIEs Are a Useful Platform to Study Intestinal Glycan Expression and the Role of Glycans in Viral Infection

### 5.1. Human Intestinal Enteroids Reflect Host Genetic Susceptibility to HuNoVs

Despite the clear association between HBGA expression and susceptibility to HuNoV infection in population studies, and structural evidence that HuNoVs bind to HBGAs, it remained unclear if HBGAs function as a receptor for HuNoV or simply as an initial attachment factor. This was because commonly used cancer-cell lines that express HBGAs, including Caco-2 cells used in binding studies, do not support productive HuNoV replication [73,74,75]. Transformed cell lines may no longer express the required protein or glycoprotein receptor. Alternatively, because transformation alters glycosylation patterns, the proper glycosylation conditions for HuNoV entry and replication may be abrogated [76]. Moreover, transformed cells have increased sialic-acid-structure expression and altered expression of sialyltransferases [77]. The increased presence of sialic acid on the cell surface could have a negative effect on HuNoV infection by masking HBGA access. Further, sialyltransferases may compete with fucosyltransferases for the same substrates, effectively reducing the presence of HBGA on the surface. Robust small-animal models for HuNoV infection are limited. This may be because they do not reflect glycosylation found in the human intestine. For example, in mice, structural characterization of O-linked glycans on gastrointestinal mucins, an abundant family of highly glycosylated secreted proteins, shows that glycan patterns vary throughout the gastrointestinal tract: in the mouse stomach, mucins display neutral O-glycans with low levels of sialylation and fucosylation, whereas in the small intestine, sialylation is dominant, with little fucosylation, and in the colon, charged fucosylated glycans prevail [78]. Although an equivalent single study of human glycan diversity of mucin glycosylation structures has not been conducted, human small intestinal mucin glycans vary from the mouse and display more fucosylated HBGA structures [78]. Recently, a zebrafish model of HuNoV infection was developed [79]. While HuNoV antigen was detected in the intestine, whether glycans act as an entry factor in zebrafish is unclear. Zebrafish share many glycan epitopes with humans but HBGAs such as A, B, Le^a^, and Le^b^ are absent [80]. HBGAs are present throughout the human gastrointestinal tract and the general expression of HBGA carbohydrate structures detected in gastric, small-intestinal, and colonic tissues has been reviewed previously [11]. Further knowledge of host glycosyltransferase expression and tissue distribution of glycans will be valuable in determining susceptibility and tropism of different gut pathogens including viruses. 

Progress in developmental and stem-cell biology led to the establishment of long-term, stable non-transformed organoid cultures from human intestinal stem cells isolated from tissue biopsies by Sato and Clevers in 2011 [81]. These multicellular cultures contain the diverse epithelial cells found in the gastrointestinal tract that are targets for intestinal pathogens including enterocytes, goblet cells, enteroendocrine cells, Paneth cells, and stem cells. Also known also as human intestinal enteroids (HIEs), these non-transformed cells are an effective cultivation system for HuNoV, allowing for new studies on virus infectivity and pathogenesis including addressing the requirement for HBGAs specific to the human intestine in infection [47,82]. We evaluated the replication of a GII.4 Sydney_2012 (TCH12-580) and a GII.3 strain of HuNoV in jejunal HIE lines derived from four secretors (J2, J3, J6, J11) and three non-secretors (J4, J8, J10). Mimicking population data, GII.4 replication was seen only in secretor HIE lines, while GII.3 replication was seen in all secretor lines and two of three non-secretor lines [47]. Subsequently, it was determined that the HIEs from GII.3-susceptible non-secretor lines (J4 and J8) were obtained from persons who were Lewis positive while the non-susceptible J10 HIE was from a Lewis-negative person. These data imply that GII.3 may bind to and infect non-secretor lines through interacting with Lewis HBGA [48,83]. 

GII.3 and GII.4 also replicate in ileal and duodenal, but not colonic secretor HIEs [47,49]. This was confirmed with studies where sets of ileal, duodenal, and colonic HIEs were generated from the same two secretor donors. Future studies are needed to determine if factors such as additional alteration of glycan expression, including the level of sialylation, altered HBGA localization or if thicker, highly O-glycosylated mucus in colonic HIE lines may affect the ability of HuNoVs to infect these lines. Thus far, matched donor jejunal with ileal/duodenal/colonic HIEs have not been generated but future studies across all segments of the small intestine and colon will provide insight on additional segment-specific differences in replication efficiency and how HBGA and glycosyltransferase expression may affect viral replication. 

### 5.2. Genetic Manipulation of HIEs Reveals FUT2 Is Necessary and Sufficient for HIE Infection by Most HuNoV Strains

**Figure 3:**
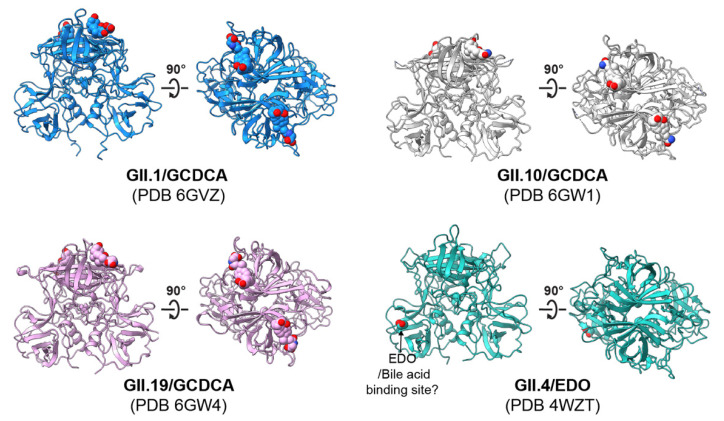
Bile-acid binding sites located on P domain dimers of GII HuNoVs. The P domains and bile acid are shown in ribbon representation and as spheres, respectively. The binding site of bile acid GCDCA on P domain dimers was reported by Kilic et al. [85]. An ethylene glycol (EDO, sphere representation) molecule binds to a GII.4 P domain at the bile acid binding site proposed by Creutznacher et al. [84]. GCDCA/EDO carbon coloration matches the ribbon diagram; heteroatoms are colored by element: oxygen, red; nitrogen, blue.

Recapitulating additional aspects of the intestinal milieu, bile or bile-acid addition is required for the replication of many HuNoV strains in HIEs [47,49,84]. While bile/bile-acid addition is essential for the replication of GII.3 (TCH04-577), GI.1 (Norwalk), and GII.17 (TCH14-38) strains, replication of GII.4 strains is only enhanced with bile addition [47,49]. X-ray crystal structures of GII.1, GII.10, and GII.19 P domains and chemical shift perturbation NMR studies of GII.4 Saga (Den_Haag_2006b) P domains indicate two different binding pockets for bile acids on the P domain (Figure 3) [43,85]. Neither site directly overlaps with the HBGA binding pocket. Yet, the addition of bile acid allows GII.1, GII.2, and GII.12 VLP binding to PGM and enhances GII.10 binding, suggesting a mechanism for bile acids enhancing infection through mediating HBGA binding [85,86]. The addition of 1% bovine bile improves the binding of three GII.2 VLPs (SMV, Chapel Hill, and Nashville) to A-blood-group saliva and PGM [87]. Interestingly, a non-secretor family was infected with GII.2 and VLPs generated from this outbreak required the presence of bile to bind to saliva from the non-secretor individuals [88]. The mechanisms by which bile acids facilitate HuNoV replication are thus far best studied for GII.3 HuNoV, a strain for which direct binding to bile acid has not been described. Mechanistic studies in HIEs determined cellular effects of bile acids permitting GII.3 replication [84]. Specifically, treatment of HIEs with the bile acid glycochenodeoxycholic acid (GCDCA), determined to be an active infection-promoting factor found in bile, leads to increased generation of cell-surface ceramides, increased endocytosis, and altered endosomal/lysosomal dynamics that facilitate GII.3 infection. The addition of GCDCA did not alter GII.3 binding to PGM. Together, these studies indicate there are strain-specific differences in how bile acids promote HuNoV infection and different strains of HuNoV may have evolved different mechanisms to utilize this readily available host factor. This would be further proof of how HuNoV uses capsid evolutionary plasticity and hijacking host factors to its advantage. 

To further investigate the requirement of FUT2 and secretor glycans in HuNoV infection, isogenic HIE lines were developed, knocking out (KO) FUT2 from a B-type secretor HIE (J2) using CRISPR-Cas9 and knocking in (KI) FUT2 in the O-type non-secretor HIE (J4) line [48]. For infectivity studies in these HIE lines, GCDCA was added to enhance (GII.4 Sydney_2012) or permit (GI.1/GII.3/GII.17) HuNoV infection. With the addition of FUT2, J4 expressed Le^b^ instead of Le^a^, indicative of functional FUT2 and FUT3 activities, and the line became permissive to GI.1, GII.3, GII.4, and GII.17 infection (Table 3). Though a receptor for HuNoV has yet to be identified, this result indicates that the receptor, or the receptor precursor, is likely present in the non-secretor J4 line but requires the addition of α1,2-fucosylation for recognition by HuNoV. Ongoing studies to identify the receptor for HuNoV infection in HIEs are discussed in Section 5.3. Conversely, KO of FUT2 from J2 resulted in loss of B Le^b^ expression and gain of Le^a^ expression, and the KO line could no longer be infected by GI.1, GII.4, or GII.17 strains [48]. The J2 FUT2 KO line remained permissive to GII.3 infection. As the J2 individual expressed FUT3, this result is consistent with epidemiological data that non-secretor, Lewis-positive individuals can be infected with GII.3 virus [89]. Inconsistent with previous results [47], GII.3 was unable to infect unmodified J4 in the presence of GCDCA. The previous studies were carried out in the presence of bile rather than GCDCA, and undetermined factors in bile may contribute to the ability of GII.3 to infect certain non-secretor, Lewis-positive lines. Replication of GII.17 and GI.1 in J4 expressing FUT2 was higher than in unmodified J2; the different ABH phenotype of these lines may account for the difference in infectivity. The J4 FUT2 KI line had an O phenotype whereas the J2 line expressed B antigen and B-blood-group is associated with a lower risk of Norwalk virus infection and inability to bind B saliva in enzyme immunoassays [39,59]. Additional genetic differences between the J4 and J2 individuals may also contribute to this difference in infection between HIE lines. Taken together, these results show that FUT2 is necessary and sufficient for replication of GII.4, GII.17, and GI.1 [48].

### 5.3. Progress towards Identification of the Fucosylated Glycan Receptor for HuNoVs

It remains to be determined if secretor HBGAs function directly as a HuNoV receptor or if a unique fucosylated glycoprotein or glycolipid is required. Prior to HIE technology, the lack of nontransformed continuous intestinal-cell-culture systems and limitations in acquiring intestinal tissue sufficient for in-depth biochemical analyses were roadblocks to glycan-receptor studies in the gastrointestinal tract. Recent and ongoing studies to characterize the glycolipid and glycoproteomic profile of susceptible and non-susceptible HIEs may guide the search for a glycosylated HuNoV receptor. Jejunal HIE studies from six different individuals show similar general lipid composition regardless of HBGA genotypes/phenotypes [83]. Approximately 80 mol % of the total lipid population is glycerophospholipids [phosphatidylcholine (PC), phosphatidylethanolamine (PE), and phosphatidylserine (PS)], free cholesterol is ~12 mol % of the lipids, and the remaining ~9% are sphingolipids [83]. Of the sphingolipids, GSLs vary between HIE lines and display HBGAs reflective of their secretor, Lewis, and ABH phenotypic profiles [83]. GII.4 Sydney_2012 VLP binds to α1,2- and α1,4-fucosylated structures such as Le ^a^ GSL and has some binding to nonfucosylated, type-1-chain precursor lactotetraosylceramide (Lc4Cer). This VLP can also bind membrane vesicles generated from GSLs isolated from two secretor, Lewis-positive HIEs, has limited binding to vesicles generated from non-secretor, Lewis-positive HIEs, and does not bind to vesicles from a non-secretor, Lewis negative individual [83]. 

**Figure 4:**
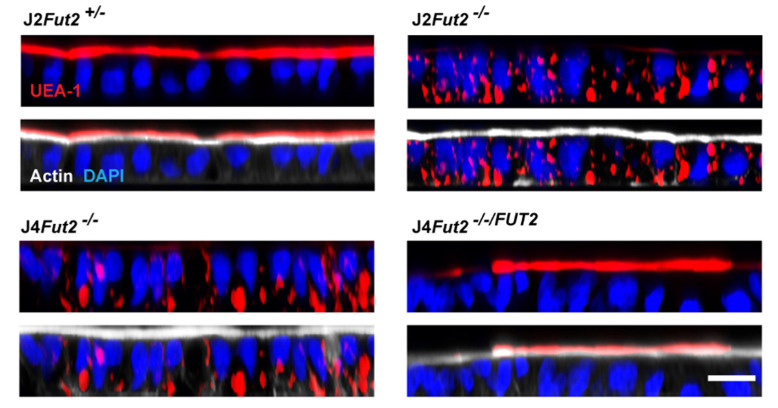
FUT2 is necessary for α1, 2-fucose expression on the apical surface. Previously unpublished images. HBGA expression was analyzed by UEA-1 lectin (red) in HIE lines as described in [48]. In all image panels, the nuclei are marked with DAPI (blue). In the bottom panels, the brush border is indicated by actin expression using phalloidin (white) for each line.

Utilizing the genetically modified HIEs developed for HuNoV studies, glycan expression in parental, KO, and KI HIEs was further characterized with immunofluorescent staining using α1,2-Fuc binding lectin, *Ulex europaeus* Agglutinin I (UEA-1). As expected, unmodified J2 HIE monolayers of polarized cells show apical UEA-1 staining, indicating that secretor HBGAs are expressed on the apical cell surface. KO of FUT2 leads to the loss of apical staining and a surprising detection of internal punctate staining (Figure 4) [48]. This pattern is recapitulated in the non-secretor J4 HIE exhibiting internal staining and J4 expressing FUT2 having strong apical staining by UEA-1. RNA sequencing analysis indicates that several fucosyltransferases in addition to FUT2 are expressed in the J2 HIE line (Table 4) and the internal staining could potentially be explained by the expression of FUT1 acting on type-2 chains [48,90]. Precedence for the role of FUT1 comes from the breast-cancer cell line T47D where FUT1 but not FUT2 controls the fucosylation of lysosomal proteins of LAMP1 and LAMP2 and lysosomal positioning [91]. Further studies using these modified HIE lines will provide insight on how cells control subcellular localization of glycoproteins through fucosylation and how pathogens hijack these processes to interact with and infect cells. Intestinal pathogens can also modulate glycan expression as seen in specific pathogen-free mice where LPS stimulates cell-surface expression of α1,2-fucosylated molecules through a FUT2-mediated mechanism [92,93]. However, bacteria are not present in the HIE cultures and are unlikely to play a role in the different fucosylation patterns seen in this model. 

**Figure 5:**
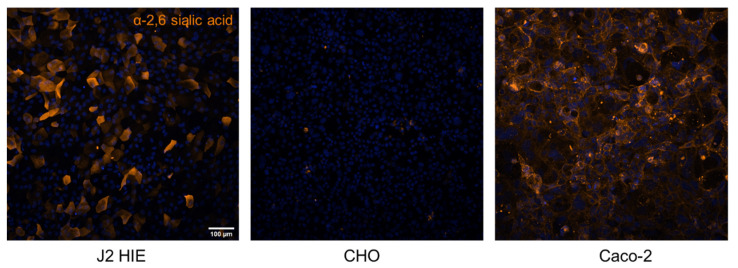
Jejunal HIE line J2 expressed α2,6-sialic acid glycans. Previously unpublished images. Monolayers of J2 (left), Chinese hamster ovary cells (CHO; middle), and Caco-2 cells (right) were fixed, permeabilized, and stained with Cy3 labeled *Sambucus nigra* lectin (orange), following protocols previously used for UEA-1 staining [48]. Caco-2 cells, that express α2,6-sialic acids, and CHO cells, that do not express α2,6-sialic acids, were included as positive and negative controls, respectively.

Of note, no sialic-acid-containing GSLs are detected from any jejunal HIE line by glycolipidomic analysis [83]. Immunofluorescent staining of J2 HIE using *Sambucus nigra* lectin, which detects α2,6-linked sialic acids, indicates some sialic acid is present on the surface of this secretor HIE (Figure 5). However, these images do not distinguish the molecule to which sialic acid is conjugated. GSLs may not be targeted for sialylation by glycosyltransferases in the jejunum. Alternatively, lipid isolation procedures may have unintentionally excluded sialylated GSLs or the levels of sialylated GSLs were simply too low for detection with chemical staining similar to what was found for sialylated GSL of epithelial cells of the adult small intestine [94]. This distinction is important to understand for future investigations on HuNoV infection and glycan expression in HIEs as there is evidence that Dijon US95/96-variant GII.4 VLPs and Chron GII.3 VLPs (derived from a GII.3 strain isolated from a chronically infected individual) can bind to sialyl Lewis x, sialyl diLewis x, and sialylated type-2-chain glycoconjugates [37,95,96,97]. Saturation transfer difference NMR revealed that VLPs derived from the GII.4 Spanish isolate Ast6139/01/Sp bind to Lewis (Le^x^, Le^y^, Le^a^, Le^b^) and to sialyl-Lewis (sLe^x^, sLe^a^) antigens; however, transfer nuclear overhauser effect spectroscopy (NOESY) indicated that the sLe^x^ neuraminic acid remains flexible, and not directly involved in the binding, when associated with the VLP [44]. Recently, an NMR study revealed that GII.4 HuNoV Saga (Den_Haag_2006b variant), MI001 (Yerseke_2006a variant), and VA387 (US95/96 variant) P domains do not bind sialoglycans [42]. However, the sialoglycans used in this study were not fucose-containing. Together these studies illustrate the plasticity of the HuNoV capsid, specifically the GII.4 HBGA binding pocket, which recognizes fucose at a minimum but has the capacity to bind Fuc, GlcNAc, and Gal residues in structurally-related glycans that also contain a terminal sialic acid.

Ultimately, GSLs expressing HBGAs may be only an initial attachment factor and the HuNoV entry receptor might instead be a protein co-receptor or a specific HBGA-displaying glycoprotein expressed by intestinal cells. Related animal caliciviruses are known to bind glycans such as sialic acid and also have confirmed proteinaceous receptors [98,99,100]. The rhesus monkey virus, Tulane virus, binds to B antigens and A-type-3 antigens in human saliva but not to antigens from O individuals [101]. The receptors for feline calicivirus (FCV), Tulane virus, and porcine sapovirus (PoSaV) are feline junctional adhesion molecule A (fJAM-A), coxsackie adenovirus receptor (CAR), and occludin, respectively [102,103,104]. These proteins are all tight-junctional proteins. CAR and occludin have known glycosylation sites and fJAM-A has a putative glycosylation site, but the exact role of glycosylation of these proteins during animal calicivirus infection needs further investigation. The MNV receptor is CD300 molecule-like family member f (CD300lf), established using CRISPR-Cas9 screens, but it is not the receptor for HuNoV replication [105,106,107]. Bone marrow-derived macrophages (BMDMs) from C57BL/6J mice are susceptible to MNV infection but BMDMs from I/LnJ mice are not. The CD300lf has altered amino-acid sequences between these two different mouse strains and is responsible for resistance to MNV infection of BMDMs from the I/LnJ mice [108]. In the susceptible C57BL/6J mouse, those amino acids contain two potential O-glycosylation sites lost in the resistant mouse. However, swapping the potential glycosylation sites in CD300lf chimeras did not alter permissiveness to MNV infection, leaving the role of glycosylation unclear. 

## 6. HBGA Interactions and Immune Response to HuNoVs

Additional evidence that HBGA interactions are critical for HuNoV infection and disease comes from findings that antibodies that block the interaction of VLPs to HBGA are a correlate of protection against HuNoV gastroenteritis [109]. Convalescent antisera from volunteers challenged with Norwalk virus blocked the binding of H-type-1, H-type- 3, and Le^b^ antigen to Norwalk VLPs [110]. Persons with HBGA-blocking antibodies prior to challenge with Norwalk virus were less likely to develop gastroenteritis and had lower viral shedding in feces than individuals who had no preexisting HBGA-blocking antibodies [111]. Until the establishment of HuNoV replication in HIEs, HBGA-blocking antibodies served as a surrogate for neutralizing antibodies. A correlation between levels of a GII.4-neutralizating antibody and HBGA-blocking titers was observed in an initial study assessing virus neutralization in HIEs with two serum samples (one with a low-HBGA-blocking titer to GII.4 Sydney_2012 and one with a high-blocking titer) [47]. A more recent study using a panel of serum samples from HuNoV VLP vaccine trials showed a strong correlation between levels of HBGA-blocking antibodies and virus-neutralization titers in HIEs [112]. In both studies, neutralizing antibody titers were higher than those measured by HBGA blocking, which may indicate the presence of other neutralizing epitopes distinct from those involved in HBGA interactions. This concept is supported by identification of a human monoclonal antibody that has neutralization activity but not HBGA-blocking activity [45]. Additionally, HBGA-blocking antibody was also a correlate of protection in an efficacy trial with a bivalent GI.1/GII.4 VLP vaccine [113].

Structural studies with an HBGA-blocking antibody show binding to the P domain of Norwalk virus but without direct contact to the HBGA binding pocket in the P domain or altering the conformation of the residues in the pocket [114,115]. In this case, steric hindrance likely explains how blocking antibodies neutralize GI.1 infection. An HBGA-blocking nanobody binds to the side of Norwalk P domain dimers and might act by altering the conformation of the P domain in a way that inhibits HBGA binding [116]. A second HBGA-blocking nanobody binds to the top of the P domain and also induces aggregation of particles, likely blocking HBGA binding through this mechanism [116]. A panel of monoclonal antibodies isolated from patients with a history of HuNoV gastroenteritis were screened for their ability to block binding of GI.3, GII.4 Sydney_2012, GII.6, and GII.17 VLPs to PGM [45]. Several of the monoclonal antibodies were capable of blocking one or more VLPs but none were able to block GII.17 binding to PGM. A specific antibody (NORO-320 IgA) was further investigated in neutralization experiments using a recombinant IgM isotype and a recombinant Fab. Both IgA and IgM NORO-320 antibodies could block GII.4 VLP binding to PGM but the Fab could not, indicating steric hindrance and VLP aggregation as the mechanism of blocking HBGA binding [45]. Interestingly, the Fab that lacked HBGA-blocking activity was capable of neutralizing GII.4 and GII.17 infection in the HIE replication system, suggesting other antiviral mechanisms in addition to steric hindrance [45]. 

Several studies using GII.4 VLPs representing viruses over decades show that GII.4 variants alter their HBGA-binding specificity over time [117,118,119]. Mice immunized with virus replicons expressing ORF2 (VP1) from GII.4 HuNoVs from different years generated antisera capable of blocking HBGA binding by temporally closer but not further-distanced VLPs [117]. Consistent with these results, GII.4 011,617 (Sydney_2012 variant) infection of HIEs was more strongly neutralized by antibodies generated against temporally closer GII.4 variants [38]. In contrast, human convalescent sera from individuals challenged with the 1968 Norwalk virus blocked HBGA binding by GI.1 VLPs not only from 1968 GI.1 but GI.1 from 2001 and GI.2, GI.3, and GI.4 (from 1999 to 2000) [118]. Alteration of an antibody epitope among GII.4 variants can also alter HBGA-binding ability. For example, an 11-residue epitope of GII.4 Den Haag_2006b is recognized by a monoclonal antibody, 3C3G3 [50]. Only two residues in this epitope vary in the GII.4 VA387 US95/96 strain (R397 and D448) but render it undetected by 3C3G3. A double mutation restored detection by 3C3G3 [50]. Interestingly, the ability of GII.4 VA387 US95/96 to bind sLe^x^ was lost with mutation at site 397 while binding to ABH antigens increased with the double mutant [50]. Evolutionary adaption of GII.4 to recognize additional HBGA types and the inability of GII.4 antisera to block HBGA binding from temporally distanced variants emerging over time is one explanation for the epidemiological predominance of GII.4 over GI.1. 

## 7. Glycan Interactions for Other Viral Pathogens

While host genetic factors in susceptibility to HuNoVs have been recognized for nearly two decades, there is now increasing evidence for HBGA–glycan interactions in other enteric viruses such as rotavirus and new evidence for respiratory viruses such as SARS-CoV-2 that are associated with gastrointestinal symptoms [7,120,121]. Rotavirus is a leading cause of severe dehydrating gastroenteritis in children under the age of 5 years worldwide. Rotavirus mediates attachment to cellular glycans through the VP8* domain of the outer capsid protein VP4 [122]. Structural and functional studies on HBGA interactions for human rotavirus are reviewed elsewhere [7,123]. These studies demonstrated that human rotavirus VP8* bind HBGA in a virus genotype-dependent manner and these interactions have implications for interspecies transmission of rotavirus strains and age-dependent restriction of infectivity for some strains [8,124,125,126,127,128,129,130]. Similar to HuNoVs, globally dominant strains of rotavirus can bind a larger repertoire of HBGAs, suggesting a similar contribution to the prevalence of some genotypes [23]. Seroprevalence studies in adults suggest higher susceptibility to rotavirus infection in secretors based on higher antibody titers compared to non-secretors [123]. However, it should be noted that the majority of the epidemiological studies examining rotavirus–HBGA interactions in children have tested samples from those presenting with acute gastroenteritis, and while there are clear rotavirus genotype-dependent differences in susceptibility to acute gastroenteritis [131,132], there is less clear evidence on whether HBGA expression influences susceptibility to asymptomatic infection. Indeed, studies in HIEs show that cultures from both secretors and non-secretors can be infected with commonly circulating rotavirus genotypes [133]. Further, in vitro infection of transformed cell lines was also independent of HBGAs expression [134]. How glycans influence disease presentation remains unclear.

HBGA interactions for other medically important gastrointestinal viruses, such as human astrovirus and sapovirus, have not been described. Studies in birth cohorts in Peru, Bangladesh, and Tanzania showed that Lewis-positive infants had a higher risk of asymptomatic adenovirus infection, but not of adenovirus-associated diarrhea. Based on these data, the most striking distinction between HuNoVs and other viruses is that HBGAs are a definitive susceptibility factor for infections with many HuNoV strains, whereas population studies on other viruses such as rotavirus and adenovirus describe HBGA associations with the presence or absence of clinical symptoms [132]. There is now new interest in HBGA interactions for SARS-CoV-2 based on several reports suggesting significantly higher susceptibility in A-blood-group individuals and reduced susceptibility among persons with the O blood group [120,121]. There is less clear evidence of association of ABO blood groups with severe outcomes of the COVID-19 disease such as acute respiratory distress syndrome, kidney injury, intubation, intensive care unit (ICU) admission, or mortality. Two biochemical studies using glycan arrays and ELISAs demonstrate the binding of SARS-CoV-2 spike protein to A-type HBGAs [135,136]. The molecular basis of SARS-CoV-2 interactions with host HBGAs is unknown and the functional significance remains to be determined. Interestingly, SARS-CoV-2 spike protein has also been shown to bind to heparan sulfate (HS), an acidic polysaccharide of the glycosaminoglycan family known to interact with several other viruses, enhancing its interaction with the angiotensin converting enzyme 2 (ACE2) receptor and facilitating infection [137,138]. Together, these studies exemplify the importance of studying host glycans as molecular mediators of virus attachment and uptake. 

## 8. Conclusions and Outstanding Questions

The importance of glycans in the binding of intestinal viral pathogens to host cells is being increasingly recognized. Physiologically relevant culture systems such as HIEs and emerging bioanalytical techniques are enabling new understanding of virus–glycan interactions. As detailed in this review, this is very well defined in the case of HBGAs and HuNoVs and the body of evidence is ever-expanding for both HuNoVs and other pathogens [139]. The vast diversity of glycan recognition by different strains of HuNoV and among GII.4 variants is indicative of the elegance of viral evolution expanding the potential host repertoire. However, there are still unresolved questions that future studies must address regarding the role of HBGAs in HuNoV infection mechanisms (Box 1). There are also outstanding questions to understand the roles of different FUT genes in intestinal glycan expression. Additionally, not every stool isolate of HuNoV tested is capable of replicating in HIEs [49,140]. Though lack of replication can be explained by low viral titer of some stool filtrates, there are higher titer isolates in stools that still fail to replicate. Most studies evaluating HuNoV replication have been carried out in an HIE line from a secretor-positive, Lewis-positive B-type individual. Use of HIE lines derived from patients with different HBGA backgrounds to screen for replication of HuNoV strains may provide clarity on HBGA-dependent differences in replication. HIEs generated from diverse individuals with different HBGA types and new analytical approaches could serve as novel tools to characterize and address key questions concerning the human intestinal glycome.

Box 1 **Key Unresolved Questions**

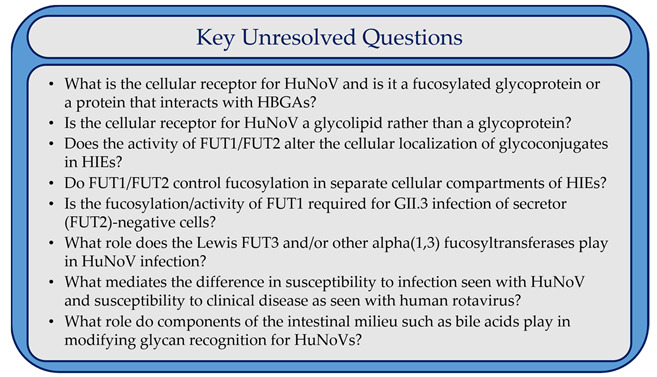

## Figures and Tables

**Table 1:** GII.4 variants.

Strain Description in Manuscript	Manuscript Reference	Type	Accession Number ^1^	RIVM Norovirus Typing Tool	Human Calicivirus Typing Tool
Dijon	[37]	VLP	AF472623	GII.4, US95/96	GII.4
GII.4 011617	[38]	Stool virus isolate	MN782359	GII.4[P16] Sydney_2012	GII.4[P16]
Grimsby (GrV)	[39,40]	VLP	AJ004864	GII.4, US95/96	GII.4
HS66	[41]	VLP	EU105469	GII.4, US95/96	GII.4
MD145	[41]	VLP	AY032605	GII.4[P4], Camberwell 1994	GII.4[P12]
MI001	[42]	P domain	KC631814	GII.4[P4], Yerseke_2006a	GII.4[P4]
PDB 4WZT	Figure 2	P domain	JX459908	GII.4[P31], Sydney_2012	GII.4[P31]
Saga	[42,43]	P domain	AB447457	GII.4[P4], Den_Haag_2006b	GII.4[P4]
Spanish isolate Ast6139/01/Sp	[44]	VLP	AJ583672	GII.4[P4], Farmington_Hills_2002	GII.4[P4]
Sydney_2012	[45]	VLP	JX459908	GII.4[P31], Sydney_2012	GII.4[P31]
TCH05	[46]	P domain	JF827296	GII.4, Hunter _2004	GII.4
TCH12-580	[47,48,49]	Stool virus isolate	Unpublished	GII.4[P31], Sydney_2012	GII.4[P31]
VA387	[39,40,50]	VLP	AY038600	GII.4[P4], US95/96	GII.4[P4]
VA387	[42]	P domain	AY038600	GII.4[P4], US95/96	GII.4[P4]

^1^ GenBank accession numbers [51]. GII.4 capsid variants were determined by two online typing tools [52,53]. P types (RNA-dependent RNA polymerase; in brackets) are listed if the accession number associated with the construct contained ORF1 information.

**Table 2:** Saliva binding patterns of HuNoV VLPs.

			Saliva Binding Pattern				
			Secretors				
Binding Group	VLP Name	Genotype (Variant)	A	B	H	Non-Secretors	Reference
1	VA387	GII.4 (US95/96)	Y	Y	Y	N	[39,40]
	Grimsby (GrV)	GII.4 (US95/96)	Y	Y	Y	N	[40]
2	MOH	GII.5	Y	Y	N	N	[39,40]
	Mexico (MxV)	GII.3	Y	Y	N	N	[40]
	Parris Island (PiV)	GII.3	Y	Y	N	N	[40]
3	BUDS	GII.2	Y	N	N	N	[40]
4	Norwalk	GI.1	Y	N	Y	N	[39,40]
	C59	GI.2	Y	N	Y	N	[40]
5	Boxer	GI.1	Y	N	Y	Y	[40]
	VA207	GII.9	Y	N	Y	Y	[39,40]
6	Operation Iraqi Freedom 031998 (OIF)	GII.21	N	N	Y	Y	[40]
7	Desert Shield virus (DSV)	GI.3	N	N	N	N	[40]
	VA115	GI.3	N	N	N	N	[40]
	Hawaii virus (HV)	GII.1	N	N	N	N	[40]

Y = yes, binds saliva. N = no, does not bind saliva.

**Table 3:** Effect of genetic modification of FUT2 expression on HuNoV infection.

HIE Line	Modification	Secretor Status	HBGA Expression	GI.1 Infection	GII.3 Infection	GII.4 Infection	GII.17 Infection
J2*Fut2*^+/−^	Parental	Positive	B Le^b^	Yes	Yes	Yes	Yes
J2*Fut2*^−/−^	*FUT2* KO	Negative	Le^a^	No	Yes	No	No
J4*Fut2*^−/−^	Parental	Negative	Le^a^	No	No	No	No
J4*Fut2*^−/−/*FUT2*^	*FUT2* KI	Positive	Le^b^	Yes	Yes	Yes	Yes

KO = knock out, KI = knock in. Data in table from [48].

**Table 4:** Expression of fucosyltransferases in J2 HIEs.

Fucosyltransferase	Activity	J2 Expression (cpm)
FUT1	Alpha(1,2)	112
FUT2	Alpha(1,2)	6587
FUT3	Alpha(1,3/1,4)	9276
FUT4	Alpha(1,3)	3411
FUT5	Alpha(1,3)	ND
FUT6	Alpha(1,3)	2485
FUT7	Alpha(1,3)	ND
FUT8	Alpha(1,6)	2534
FUT9	Alpha(1,3)	47
FUT10	Alpha(1,3) putative	715
FUT11	Alpha(1,3) putative	1568

cpm = counts per million. ND = not detected. RNA sequencing data are deposited as described in Lin et al. [90].

## Data Availability

All data are present in the main text or available from the authors upon request.
